# Comparative analysis of full genomic sequences among different genotypes of dengue virus type 3

**DOI:** 10.1186/1743-422X-5-63

**Published:** 2008-05-21

**Authors:** Chwan-Chuen King, Day-Yu Chao, Li-Jung Chien, Gwong-Jen J Chang, Ting-Hsiang Lin, Yin-Chang Wu, Jyh-Hsiung Huang

**Affiliations:** 1 Institute of Epidemiology, College of Public Health, National Taiwan University, Taipei, Taiwan(10020), PRoC; 2 Institute of Veterinary Public Health, College of Veterinary, National Chung-Shin University, Taipei, Taiwan(402), PRoC; 3 Center for Disease Control, Department of Health, Taipei, Taiwan (100), PRoC; 4 Division of Vector-Borne Infectious Diseases, National Center for Infectious Diseases, Centers for Disease Control and Prevention (CDC), Fort Collins, Colorado (80521), USA

## Abstract

**Background:**

Although the previous study demonstrated the envelope protein of dengue viruses is under purifying selection pressure, little is known about the genetic differences of full-length viral genomes of DENV-3. In our study, complete genomic sequencing of DENV-3 strains collected from different geographical locations and isolation years were determined and the sequence diversity as well as selection pressure sites in the DENV genome other than within the E gene were also analyzed.

**Results:**

Using maximum likelihood and Bayesian approaches, our phylogenetic analysis revealed that the Taiwan's indigenous DENV-3 isolated from 1994 and 1998 dengue/DHF epidemics and one 1999 sporadic case were of the three different genotypes – I, II, and III, each associated with DENV-3 circulating in Indonesia, Thailand and Sri Lanka, respectively. Sequence diversity and selection pressure of different genomic regions among DENV-3 different genotypes was further examined to understand the global DENV-3 evolution. The highest nucleotide sequence diversity among the fully sequenced DENV-3 strains was found in the nonstructural protein 2A (mean ± SD: 5.84 ± 0.54) and envelope protein gene regions (mean ± SD: 5.04 ± 0.32). Further analysis found that positive selection pressure of DENV-3 may occur in the non-structural protein 1 gene region and the positive selection site was detected at position 178 of the NS1 gene.

**Conclusion:**

Our study confirmed that the envelope protein is under purifying selection pressure although it presented higher sequence diversity. The detection of positive selection pressure in the non-structural protein along genotype II indicated that DENV-3 originated from Southeast Asia needs to monitor the emergence of DENV strains with epidemic potential for better epidemic prevention and vaccine development.

## Background

Dengue fever (DF) and its more severe forms, dengue hemorrhagic fever (DHF) and dengue shock syndrome (DSS), have emerged as major public health problems in tropical and subtropical areas [1,2]. Infection with dengue viruses (DENV), which are maintained in a human-mosquito transmission cycle involving primarily *Aedes aegypti *and *Aedes albopictus*, can result in various clinical manifestations ranging from asymptomatic to DF, DHF, DSS and death [3]. The occurrences of dengue epidemics in the past 30 years have been characterized by the rising incidence rates of infection and continuous expansion in geographic distribution of DHF epidemics [4]. Importantly, the epidemics of DHF have become progressively larger in the last 20 years in many dengue endemic countries [5]. The increasingly widespread distribution and the rising incidence of DF and DHF are related to increased distribution of *A. aegypti*, global urbanization and rapid and frequent international travel.

Epidemiological analysis reveals that some DENV strains are associated with mild epidemics with low occurrences of DHF cases and inefficient virus transmission, whereas others are more likely to cause severe epidemics with high incidence of DHF/DSS and rapid virus transmission [6,7]. The large DHF epidemics in Indonesia in the 1970s and Sri Lanka after 1989 provided evidence supporting this phenomenon [8,9]. Dengue virus serotype 3 (DENV-3) re-appeared in Latin Americain 1994 after its absence for seventeen years. The virus was detected initially in Panama and soon dispersed throughout Central and South America during the following years [10,11]. This introduction coincided with an increased number of DHF cases in this region. Although the genotype originating in Southeast Asia has been postulated as the major cause of the increased virulence, the molecular marker associated with a difference in virulence among genotypes at the full-genomic level is still largely unknown.

Dengue is caused by four antigenically related but genetically distinct viruses (DENV-1, -2, -3 and -4) belonging to the genus *Flavivirus*, family *Flaviviridae *[12]. DENV is a single stranded, positive-sense RNA virus, approximately 10,700 nucleotides in length. The genome contains a single open reading frame (ORF) that encodes a polyprotein, which is co- and post-translationally processed to produce three structural proteins, including capsid (C), pre-membrane (prM) and envelope (E), and seven nonstructural (NS) proteins (NS1, NS2A, NS2B, NS3, NS4A, NS4B and NS5) [12,13]. A considerable number of studies have revealed that each serotype of DENV is composed of phylogenetically distinct clusters that have been classified into "genotypes" or "subtypes," and each genotype is also composed of phylogenetically distinct "groups" or "clades." A previous study has classified DENV-3 strains into four genotypes based on limited numbers of nucleic acid sequences from the prM and E protein genes [6]; DENV-3 strains have also been re-classified into five genotypes [14]. Growing evidence suggests the existence of DENV strains with different epidemic potentials. This evidence is supported by the following observations: (1) the differences in fitness among various genotypes of DENV-2 reflect their different replication capabilities in human monocytes and dendritic cells [15]; (2) around 1991, clade replacement among DENV-3 genotype II containing isolates from Thailand was associated with changing serotype prevalence and incidence of DHF epidemics [16]; and (3) sudden changes in the genotype of DENV at a single locality have been observed that appeared to originate from the genetic bottleneck of a large viral population [14,17]. This sudden genotype replacement has been associated with more severe DHF epidemics in Indonesia and Sri Lanka [9,18]. However, most of these studies involved the E gene alone. This raises an important question: Is the introduction of different DENV genotypes in disparate geographical locations a result of sequence differences outside of the E gene altering their epidemic potential, or it is simply a stochastic event in viral evolution?

Dengue epidemics in Taiwan are usually initiated by imported index cases (King et al., 2000). The re-emergence of dengue outbreaks in Taiwan started when DENV-2 was re-introduced into the off-islet of Hsiao-Liu-Chiu in 1981. In 1987–1988, another large-scale DENV-1 outbreak occurred in Kaohsiung and Pingtung in southern Taiwan [19]. Although DENV-3 was detected sporadically from imported index cases, no DENV-3-related epidemic occurred until 11 DHF cases were confirmed in Kaohsiung in 1994 and 23 DHF cases in Tainan in 1998 [20]. Taiwan neighbors many Southeast Asian countries and more than 25,000 travelers visit these adjacent countries annually. The surveillance system implemented by the Center for Disease Control in Taiwan (Taiwan-CDC) routinely detects many imported dengue cases each year. Thus, Taiwan is an ideal place to study the evolution and dispersion of DENV that may have different epidemic potential, particularly in the 1994 and 1998 DHF epidemics in Taiwan that coincided with the DHF epidemics in Southeast Asian countries [21]. Complete genomic sequencing of DENV-3 strains collected from different geographical locations and isolation years offers the opportunity to understand the genetic stasis and possible selection pressure sites in the DENV genome other than within the E gene.

## Methods

### Sources of DENV-3 viruses

The blood samples of suspected dengue patients, obtained from the sentinel hospitals/clinics located in Tainan, Kaohsiung and Pingtung in southern Taiwan, were sent to the Infectious Disease Epidemiology Laboratory at National Taiwan University (NTU) and Taiwan-CDC for laboratory confirmation. The study protocol was approved by the College of Public Health Research Human Subject Ethics Review Committee at NTU. A suspected and confirmed dengue case was defined as previously described and confirmed by both laboratories [20,22]. Imported and indigenous dengue cases were defined based on the patients' travel history to dengue-endemic or -epidemic countries within 3–14 days before the onset of the disease.

Due to few DENV-3 epidemics and limited DENV-3 isolates identified before 1998 in Taiwan, we focused our study on comparing the sequences of different DENV-3 isolates in 1998 and considering various epidemiological characteristics, including temporal, geographical and host factors. Six DENV-3 isolates were selected for full-length sequencing: (1) an isolate from the imported DENV-3 infected case in 1998; (2) an isolate from the indigenous DF and DHF cases during the 1998 epidemic in Tainan, Taiwan; (3) the 1998 isolate from a geographical location in Tainan other than the 1998 epidemic area; (4) an isolate from the same geographical location as the 1998 Tainan's epidemic but in 1999; and (5) an isolate from indoor mosquitoes during the 1998 dengue/DHF epidemic in Tainan. The epidemiological characteristics of these six DENV-3 isolates are summarized in Table 1, and their GeneBank accession numbers are DQ675520–DQ675533. In addition to the 1998–99 DENV-3 strains, four local isolates obtained from Taiwan during previous years, kindly provided by Taiwan-CDC, were also used for comparison, including four strains isolated from indigenous DF patients during the 1994–95 epidemic in Kaohsiung [94TWKH33 (Accession No.: DQ675534), 94TWKH65 (Accession No.: DQ675535), 94TWKH25 (Accession No.: DQ675536), 95TW466 (accession No.: DQ675519)]. Isolate 95TW466 with low passage history (two passages in C6/36 cells) was subjected to full-length genomic sequencing together with the above six isolates from 1998–99, constituting seven full-length DENV-3 sequences from Taiwan. The remaining three 1994 DENV-3 isolates were sequenced only from the 5' NCR to the COOH-terminus of the E gene region for phylogenetic analysis.

**Table 1:** Characteristics of the full-length genome sequences of the DENV-3 isolates investigated in this study

Geographic origin	Disease Status^a^	Year	Strain	Genotype	Passage history^b^	GenBank accession no
Philippines	?	1956	H87	V	C6/36, SMB	M93130
Guangxi China	?	1980	80-2	V	?	AF317645
Thailand	DF	1994	C0360/94	II	?	AY923865
Thailand	DHF	1994	C0331/94	II	?	AY876494
Indonesia, Jakarta	DF	2004	TB55i	I	?	AY858048
Indonesia, Jakarta	DF	2004	TB16	I	?	AY858047
Indonesia, Jakarta	DF	2004	PI64	I	?	AY858046
Indonesia, Jakarta	DF	2004	PH86	I	?	AY858045
Indonesia, Jakarta	DF	2004	KJ71	I	?	AY858044
Indonesia, Jakarta	DF	2004	KJ46	I	?	AY858043
Indonesia, Jakarta	DF	2004	KJ30i	I	?	AY858042
Indonesia, Jakarta	DF	2004	FW06	I	?	AY858041
Indonesia, Jakarta	DF	2004	FW01	I	?	AY858040
Indonesia, Jakarta	DF	1998	den3_98	I	?	AY858039
Indonesia, Jakarta	DF	1988	den3_88	I	?	AY858038
Indonesia, Jakarta	DF	2004	BA51	I	?	AY858037
Indonesia	Vaccine candidate		Sleman/78	I	?	AY648961
Singapore	unknown	1995	Singapore 8120/95	II	?	AY766104
Indonesia, Sumatra	DF	1998	98902890	I	?	AB189128
Indonesia, Sumatra	DHF	1998	98901517	I	?	AB189127
Indonesia, Sumatra	DSS	1998	98901437	I	?	AB189126
Indonesia, Sumatra	DSS	1998	98901403	I	?	AB189125
Brazil	DSS	2002	BR74886/02	III	?	AY679147
Martiniquw	?	1999	D3/H/IMTSSA-MART/1999/1243	III	?	AY099337
Sri Lanka	?	2000	D3/H/IMTSSA-SRI/2000/1266	III	?	AY099336
Taiwan(Kaoshiung)	DF	1995	95TW466	I	AP61 2, C6/36 1	In this study
Taiwan Indonesia-imported	DF	1998	98TW182	II	C6/36 1	In this study
Taiwan (Pingtung)	DF	1998	98TW358	II	C6/36 1	In this study
Taiwan (Tainan)	DF	1998	98TW364	II	C6/36 1	In this study
Taiwan (Tainan)	DHF	1998	98TW368	II	C6/36 1	In this study
Taiwan (Tainan)	mosq	1998	98TWmosq	II	C6/36 1	In this study
Taiwan (Tainan)	DF	1999	99TW628	III	C6/36 1	In this study

a. ? indicates no information available about the disease status of the patient from which the virus was isolated.

b. ? indicates no information available about the passage history of the virus strains. The C6/36 or AP61 number indicates that the virus strain was obtained after the noted number of passages in a C6/36 or AP61 mosquito cell line infected with the original patient's plasma sample. SMB indicates suckling mice brain inoculation.

### Viral RNA extraction, RT-PCR and nucleotide sequencing

Acute-phase serum or plasma samples collected from the dengue patients within seven days after the onset of fever were used for both virus isolation and molecular diagnosis [23,24]. Molecular diagnosis by reverse transcriptase polymerase chain reaction (RT-PCR) amplification and subsequent nucleic acid sequencing was performed as previously described, and a complete list of the PCR and sequencing primers utilized is available upon request [25]. The RNA genomic 5' and 3' terminal 20 nucleotide sequences were not confirmed independently and were assumed to be of the same length and sequence as the prototype strain H87 in this study.

### DENV-3 Viral Sequence and Phylogenetic analysis

A total of 25 complete genomic sequences of DENV-3 strains and one DENV-1 strain A88 (GenBank accession number AB074761) were aligned using the multiple sequences alignment ClustalX [26]. These sequences were further combined with all available sequences of the complete E gene or the complete prM and partial E genes (to nucleotide position 1140 of the E gene) of DENV-3 deposited in the GenBank database at the National Center for Biotechnology Information (NCBI). Therefore, the complete E gene (1479 nt) dataset consisting of a total of 168 sequences and the prM and partial E gene (705 nt) dataset of a total of 195 sequences were used for phylogenetic analysis. A complete list of the sequences along with associated epidemiological information is available upon request.

The percentage of sequence similarities and differences were calculated using Bioedit v3.6 program [27]. Pairwise comparisons of both nucleotide and amino acid sequences of DENV-3 isolates were performed using the program MEGA v3.1 (Molecular Evolutionary Genetics Analysis, Pennsylvania State University, PA) to determine the mean and range of the proportional difference (p-distance) [28]. The model of nucleotide substitution that best described DENV-3 sequence evolution was identified using the program Modeltest 3.0 [29]. The resulting most complex GTR+I+Γ substitution model (general time reversible model, GTR, a proportion of sites modeled as invariant, I, variation in rates among sites modeled using the gamma distribution, Ã) was selected to be the best fit to the data using the hierarchical likelihood ratio tests (hLRTs) and Akaine information criterion (AIC). The estimated parameter values from this model were as follows: relative substitution rates among nucleotides were A ↔ C = 1.6120, A ↔ G = 9.5789, A ↔ T = 1.7255, C ↔ G = 0.6272, C ↔ T = 29.7738, G ↔ T = 1.0; proportion of invariable sites (I) was 0.4475; gamma distribution of among-site rate variation (Ã) was 1.2293; and estimated base composition of A = 0.3268, C = 0.2145, G = 0.2539, and T = 0.2048. A maximum likelihood (ML) tree using these parameter settings was estimated using the DNAML in Phylip v3.6 package [30]. Bootstrap analysis with 1,000 re-samplings was used to determine confidence values for groupings within the phylogenetic tree. In addition, a posterior probability distribution tree, generated by implementing the recently developed Bayesian hierarchical phylogenetic model utilizing a Metropolis-coupled Monte Carlo Markov Chains (MC)^3 ^algorithm in the MrBayes program (version 3.1, [31]) was compared with the evolutionary tree of DENV-3 generated by the ML method. Indeed, the Bayesian approaches for constructing phylogenetics have several advantages. First, the primary analysis often provides faster estimates of the tree and measurements than the estimates obtained using ML bootstrapping techniques. Secondly, Bayesian model selection offers advantages over likelihood methods in that the competing evolutionary hypotheses need not to be nested, and it does not rely on standard likelihood assumptions. In other words, the starting trees in Bayesian method are randomly chosen, and multiple runs of the same dataset are generally made with different starting trees to check convergence of the process. The programs' default settings for prior probability were used in our analysis. Bayesian Markov Chain Monte Carlo (BMCMC) processes, considering the heterogeneity in the evolutionary process and thus incorporating a discrete gamma distribution of four classes of substitution rates across mutation sites, were run for 500,000 generations. Output trees were sampled every 100 generations but the first 1,000 trees were discarded before the process reached the convergence state. The resulting trees were rooted using a DENV-1 strain A88 isolate as described.

To analyze the selection pressure in DENV-3, the CODEML program from the PAML package was employed by implementing a maximum-likelihood method. This method presents major advantages over simpler pairwise comparisons in considering the transition/transversion rate bias, non-uniform codon usage, and phylogenetic relationships among the sequences [32]. Positive selection at a small number of codons can be detected by comparing various models of codon evolution which differ in how the rates of synonymous (dS) and nonsynonymous (dN) substitutions (denoted as ω) are treated among codons or within lineages using likelihood ratio tests. To analyze selection pressures at individual codons, we compared the M7 and M8 model. In the M7 model, 10 categories were assigned and estimated from the data, which specified only neutral evolution; however, the M8 model allowed positive selection by adding an 11^th ^codon category at which dN/dS can exceed 1.0. To examine selection pressures along the lineages, the free ratio model, which allows certain lineages to have ω ratios different from the background, was implemented in the M3 model. Additionally, parameters involving the incorporation of classes of codons where ω >1 were used by comparing the value of the likelihood from M0, in which the specified neutral evolution of ω is constrained to be equal to or less than 1 at all codons among all lineages. The comparison was again assessed using the likelihood ratio test. If positive selection was found, the Bayesian method was applied to identify the specific codon that may have been subjected to positive selection pressure.

## Results

### Comparison of full-length nucleotide and amino acid sequences among DENV-3 strains from Taiwan

We have determined the complete nucleotide sequences (10,707 nucleotides in length with an ORF of 3,390 amino acids) of the seven different DENV-3 strains from Taiwan (Table 1). The percentages of nucleotide and amino acid identities of the entire ORF among these strains, compared with the prototype DENV-3 strain H87 isolated in the Philippines in 1956, are shown in Table 2. The indigenous DENV-3 isolates from the 1998 epidemic area in Tainan City (98TW364 and 98TW368) and from the sporadic case in Pingtung (98TW358) displayed the highest similarity, with 99.9% sequence identity in both nucleotide and amino acid sequences. The 1998 imported 98TW182 strain showed slightly lower nucleotide and amino acid sequence identity (98%) relative to these 1998 indigenous Taiwanese DENV-3 isolates. The DENV-3 isolates of Taiwan from years other than 1998, including the 1995 Kaoshiung 95TW466 and the 1999 Tainan 99TW628 strains, showed higher sequence diversity compared with the 1998 DENV-3 Taiwan isolates (94% nucleotide and amino acid sequence identity), which suggested that they might have originated from different countries. Further phylogenetic analysis revealed that these viruses belong to different genotypes (Genotype I and III; see the section ''Phylogenetic analysis of DENV-3'' for details).

**Table 2:** Percentage identity within the entire genome of seven different DENV-3 obtained from Taiwan, as compared with the prototype strain H87.

		pairwise nucleotide identity (%)							
	Strain	1	2	3	4	5	6	7	8

1	H87	-	95.1	95.0	95.0	95.0	95.3	94.9	95.0
2	98TW182	95.0	-	98.0	98.0	98.0	94.2	94.1	98.0
3	98TW358	95.0	98.0	-	99.9	99.9	94.0	94.2	99.9
4	98TW364	95.0	98.0	100.0	-	99.9	94.0	94.2	99.9
5	98TW368	95.0	97.9	99.9	99.9	-	94.0	94.2	99.9
6	95TW466	95.3	94.0	93.8	93.8	93.8	-	93.9	94.0
7	99TW628	94.8	93.9	94.0	94.0	94.0	93.8	-	94.2
8	98TWmosq	94.9	97.9	99.9	99.9	99.9	93.8	94.0	-

		pairwise amino acid identity (%)							

The upper-right matrix corresponds to nucleotide sequences and the lower-left matrix to the amino acid sequences.

Compared to the prototype strain H87, several unique amino acid substitutions that serve as unique signature sites for each genotype were found within the full genomic sequences of the selected DENV-3 isolates from Taiwan or other countries and are listed by the order of the gene in Table 3. Among those, several substitutions changed the polarity, charges, or hydrophobicity of these amino acids, which were present only in genotype III of DENV-3, including the change from threonine (T) to alanine (A) at position 112 of the C region, leucine (L) to histidine (H) at position 55 of the prM region, L to T at position 301 of the E region, isoleucine (I) to T at position 115 of the NS3 region, and lysine (K) to T and aspartic acid (D) to asparagine (N) at positions 585 and 835 of the NS5 region. Similar signature sites experiencing amino acid property alterations in genotype II included a change from T to A at position 57 of the prM region, L to serine (S) at position 178 of the NS1 region, and A to T at position 133 of the NS2A region. Thus, our data suggested that different genotypes of DENV-3 experience different amino acid changes at both structural and non-structural genes, and the sites of these substitutions could serve as signature sites for genotype identification.

**Table 3:** Description of amino acid differences among the selected DENV-3 from Taiwan and other countries as compared to reference strain H87

Geno	V	V	I	I	I	I	II	II	II	II	II	III	III	III	III
Strain*	H87	80-2	Ind88	Ind04	TW95	Ind98	Tha94	Sing95	Tw182	Tw358	Tw368	TW99	Martini	SriLan	Brazil

Year	56'	80'	88'	04'	94'	98'	94'	95'	98'	98'	98'	99'	99'	00'	02'

**Capsid**															
35**	R									K	K	K	K	K	K
65	V		I	I		I									
82	K		R	R	R	R									
97	K		R	R	R	R								Q	
108	M							I				I	I	I	I
112	T											A	A	A	A
**prM**															
55	H		L	L	L	L	L	L	L	L	L				
57	T						A	A	A	A	A				
128	L		F	F	F	F									
**Envelope**															
68	I		V	V	V	V	V								
81	I											V	V	V	V
124	S			L		L	P	P	P	P	P	P	P	P	P
132	H									Y	Y	Y	Y	Y	Y
154	E						D	D	D	D	D				
160	A						V	V	V	V	V				
169	A			V	V	V		V	V	V	V	H	T	T	T
231	R			K	K	K									
270	T						N	N	N	N	N	N	N	N	N
301	L			S		S						T	T	T	T
303	T		A	A	A	A									
383	K											N	N	N	
452	I											V	V	V	V
479	A			V	V	V	V			V	V				
**NS1**															
178	L						S	S	S	S	S				
188	V						I	I	I	I	I				
217	L						F	F	F	F	F				
339	N											S	S	S	S
**NS2A**															
37	L											F	F	F	F
55	H		R	R		R									
133	A						T	T	T	T	T				
153	T		M	M	M										
175	I						V	V	V	V	V	V	V	V	V
**NS3**															
115	I											T	T	T	T
255	R		K	K	K	K									
324	D						E	E	E	E	E				
350	E		D	D		D									
356	V		A	A		A						A	A	A	A
452	V						A	A	A	A	A	A	A	A	A
589	K						R	R	R	R	R	R			
**NS4A**															
89	I		V	V	V	V									
99	D											E	E	E	E
148	V											I	I	I	I
115	V											I	I	I	I
190	L		F	F	F	F									
**NS4B**															
116	V											I	I	I	I
191	L		F	F	F	F									
**NS5**															
281	K			R	R	R									
288	S											N	N	N	N
336	M		T	T	T	T									
422	R			K								K	K	K	K
585	K											T	T	T	T
619	I						V	V	V	V	V	V		V	
749	R		K	K	K	K	K	K	K	K	K				
835	D											N	N	N	N
876	N						D	D	D	D	D				

*The GeneBank accession numbers for the strains of DENV-3 compared are H87: M93130; 80-2: AF317645; Ind88: AY858038; Ind04: AY858040; TW95: DQ675519; Ind98: AY858039; Tha94: AY923865. Sing95: AY766104; TW182: DQ675520; TW358: DQ675522; TW368: DQ675525; TW99: DQ675533; Martini: AY099337; SriLan: AY099336; Brazil: AY679147.

**A blank cell indicates an amino acid identical to that of strain H87.

### Phylogenetic analysis of DENV-3

**Figure 1:**
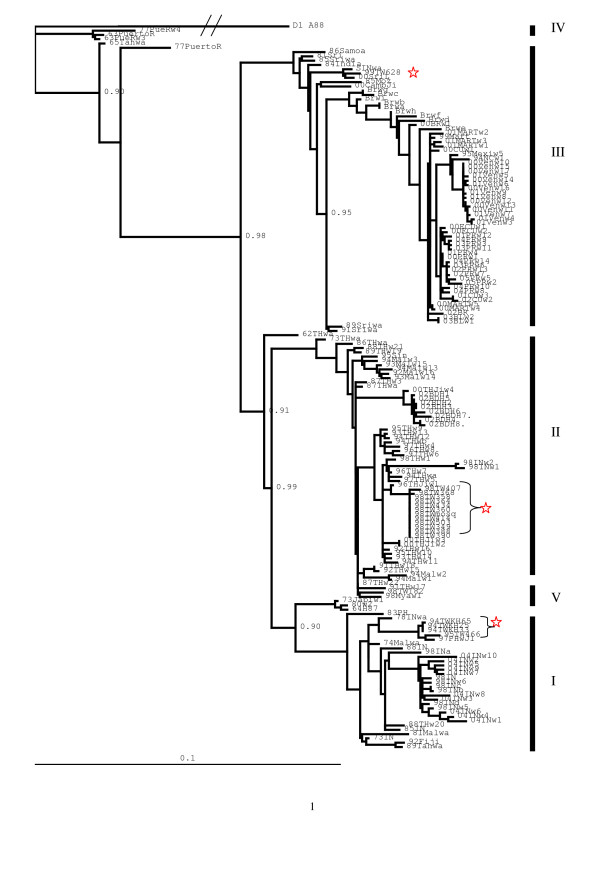
**The Bayesian hierarchical consensus tree showing the phylogenetic relationships between DENV-3 genotypes is based on the complete E gene sequences (1479 bp) from the 168 DENV-3 strains sampled globally. **The names of the DENV-3 isolates refer to the year of isolation and the country of origin. In cases where there is more than one isolate from a given country and year, a unique isolate number (or code) is also given. The abbreviations of the names of the countries are: Bangladesh (BD), Bolivia (BL), Brazil (Br), Cambodia (Cam), Cuba (Cu), Ecuador (ECU), Indonesia (Indo), Japan (Jap), Martinique (Mart), Mexico (Mexi), Mozambique (Moza), Malaysia (Mal), Myanmar (Mya), Nicaragua (Nic), Peru (PR), Puerto Rico (PueR), Philippines (PH), Singapore (Sin), Sri Lanka (SriL), Tahiti (Tah), Thailand (TH), Taiwan (TW), Venezuela (Ven). Bootstrap values greater than 0.9 based on Bayesian posterior probabilities are shown for key nodes. The major genotypes of DENV-3 are also labeled. The tree was rooted using DENV-1 strain A88 (GenBank accession number: AB074761) as the outgroup. Taiwan DENV-3 isolates are marked with a star.

The phylogenetic trees of DENV-3 were constructed from the two different nucleic acid dataset alignments: (1) partial sequences of the prM and E gene region (prM/E) from 10 isolates obtained from Taiwan and 185 sequences available from GenBank; (2) complete E gene sequences including 168 isolates from both Taiwan and GenBank. The trees derived from the maximum likelihood method and the Bayesian method based on both datasets were very similar to each other. Thus, only the posterior probability tree derived from the Bayesian method based on the complete E gene sequences is shown (Fig 1). The DENV-3 strains isolated in Taiwan during the 1994–1995's outbreak were grouped into genotype I, together with the earlier DENV-3 strains from Southeast Asia, including those from Indonesia, Malaysia, the Philippines and the South Pacific islands. However, all the DENV-3 strains isolated during the 1998 dengue/DHF epidemic in Taiwan were classified as genotype II, which consists mainly of viruses from Thailand. Interestingly, the only DENV-3 strain (98TW182) examined that was imported to Taiwan from Indonesia in 1998 did not cluster with the other Indonesia DENV-3 isolates. It is related closely to the isolate from Myanmar from 1998, which grouped with the Thailand isolates into genotype II. Genotype III of DENV-3 consists of the strains from Sri Lanka, India, Africa and Samoa that were recently introduced into Central and South America and caused major DHF epidemics in many countries. The 99TW628 strain, isolated in 1999 from Tainan in Taiwan, belongs to this genotype. Genotype IV, representing the earlier American genotype, consists of the isolates from Puerto Rico in 1963/77 and Tahiti in 1965, and viruses belonging to this genotype have not been isolated since the 1970s. Genotype V consists of the 80-2 strain isolated from China in 1980, the H87 strain isolated from the Philippines in 1956, and the Japanese isolate from an imported case in 1977.

### Sequence divergence in nucleotide and amino acid sequence among various regions of full-length sequences of different genotypes of DENV-3

With the lack of full-length sequences of viruses belonging to old American genotype IV, only four DENV-3 genotypes, including representatives of genotype I (98TW366), genotype II (98TW349), genotype III (98TW628) and genotype V (H87) were compared. The sequence divergences in nucleotide and amino acid were calculated as the p-distance by adjusting the lengths of different genes [25,33]. The highest nucleotide diversity was found in the NS2A gene (mean ± SD: 5.84 ± 0.54), followed by the E gene (mean ± SD: 5.04 ± 0.32). Similar results were observed for amino acid diversity, which was also the highest in the capsid gene (mean ± SD: 3.13 ± 1.15), followed by the NS2A gene (mean ± SD: 2.57 ± 0.62) (Table 4).

**Table 4:** Comparison of sequence diversity (p-distance, %) of full-length genomic sequences among different genotypes of dengue virus type 3

	Capsid	prM	E	NS1	NS2a	NS2b	NS3	NS4a	NS4b	NS5
**nucleotide**	3.24 ± 0.54	4.37 ± 0.52	5.04 ± 0.32	4.37 ± 0.39	5.84 ± 0.54	4.02 ± 0.59	4.55 ± 0.30	4.21 ± 0.54	3.85 ± 0.41	4.23 ± 0.23
**Amino acid**	3.13 ± 1.15	1.41 ± 0.53	1.60 ± 0.34	1.54 ± 0.36	2.57 ± 0.62	0.54 ± 0.18	0.99 ± 0.24	1.33 ± 0.53	0.85 ± 0.31	1.17 ± 0.20

### Analysis of selection pressure among different viral regions of DENV-3 full-length sequences

**Figure 2:**
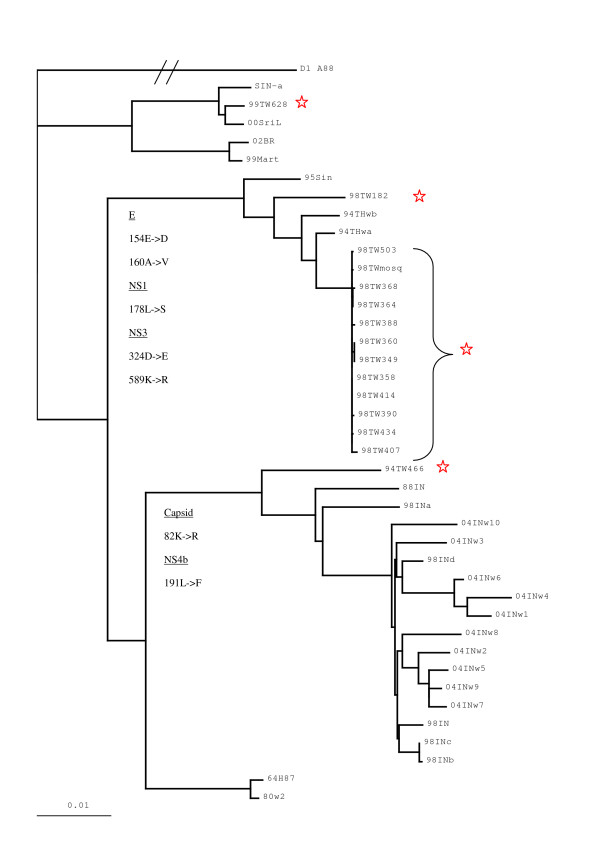
**The Maximum likelihood phylogenetic tree shown here is based on the complete genomic sequences of 25 DENV-3 strains available from GenBank.** The tree was rooted using DENV-1 strain A88 (GenBank accession number: AB074761) as the outgroup. The major amino acid changes along lineages within genotype I and II are also labeled. Taiwan DENV-3 isolates are marked with a star.

To determine whether higher sequence diversity in certain genes could be the result of natural selection pressures, we implemented the M7 and M8 selection models to determine whether positive selection pressure among all codons from the full-length DENV-3 sequences could be detected by using the CODEML program from PAML [32]. The results suggested that both structural and non-structural genes of DENV-3 were under neutral selection. Although the E gene showed positive selection (ω = 2.15) with statistical significance (p = 0.01) when using the larger dataset with 73 sequences, no specific site with positive selection could be detected. To further examine the selection pressure along the lineage, genotype I, II, III and V, based on the phylogenetic tree of the full-length sequences (Fig 2), were examined separately using the M3 model. The results are summarized in Table 5. Although there were positive selection pressures detected in the C and NS4B genes of genotype I, and in the E, NS1 and NS3 genes of genotype II, only the NS1 gene of genotype II showed statistically significant positive selection pressure. Furthermore, positive selection was detected at position 178 of the NS1 gene (substitution of S for L).

**Table 5:** Positive selection and relevant parameter values among different genomic regions of full-length DENV-3 sequences

Gene	dN/dS			
	
	Genotype I	Genotype II	Genotype III	Genotype V
Capsid	**5.68**	0.00001	0.05	0.00001
prM	0.00001	0.14	0.00001	0.00001
E	0.02	**999**	0.03	0.097
NS1	0.00001	18.2*	0.02	0.66
NS2A	0.00001	0.00001	0.02	0.08
NS2B	0.00001	0.00001	0.00001	0.00001
NS3	0.008	**999**	0.007	0.04
NS4A	0.04	0.00001	0.08	0.00001
NS4B	**19.37**	0.00001	0.00001	0.1
NS5	0.00001	0.008	0.018	0.00001

*p < 0.05 with statistical significance

### Changes at the 5' and 3' non-coding regions (NCR) and secondary structure analysis

Changes occurring in the 5' NCR and 3' NCR were examined among the DENV-3 viruses isolated in Taiwan and other countries. In the 5' NCR, positions 62, 90, 109 and 112 had nucleotide changes that were distinguishable for the specific genotype. Among them, a G to A change at position 62 was frequently seen in genotype I, a C to T change at position 90 and a G to A change at position 109 were observed only in genotype II, and an A to G change at position 112 was present in genotype III. Interestingly, there was consistently an additional 11-nucleotide sequence, AGTGAAAAAGA, inserted in the 3' NCR close to the end of the open-reading frame (ORF) of the DENV-3 strains isolated in recent years, compared to the prototype strain H87. In the 3' NCR, nucleotide changes at position 111, 129, 220 and 438 (nucleotide numbering beginning at 5'-terminus of 3' NCR after the stop codon) were observed from the strains circulating recently, which differed from the strain H87. However, none of these changes had any effect on the predicted secondary structure of the 3' NCR RNA (data not shown). The putative genome cyclization sequence UCAAUAUG, located between nucleotides 38 and 46 of the C gene, was conserved in all DENV-3 viruses.

## Discussion and conclusion

Viral sequence comparisons among isolates from dengue epidemics of different disease severities may provide valuable information regarding the molecular basis of the epidemic potential of the virus. DENV-3 re-appeared in 1998 in Taiwan and caused the DF/DHF epidemic in Tainan City after its first introduction in 1994 [20]. This stimulates a great interest in understanding the molecular relationship of DENV-3 isolates in Taiwan during inter-epidemic periods and in comparing them with the strains circulating globally to understand evolutionary trends and geographical expansions. Here, we confirmed that the dengue epidemics in Taiwan were strongly associated with the globally circulating DENV-3 due to constant introduction of viruses from Southeast Asia by Taiwanese travelers. Our data demonstrates the sequence diversity among the full-genomic sequences of DENV-3 and the positive selection pressures exerted in different lineages (i.e. genotypes) at sites in DENV-3 non-structural genes.

Since most Taiwan dengue epidemics were initiated by the introduction of virus from imported cases [21], phylogenetic analysis provides essential information to understand the history and origin of all Taiwan DENV-3 isolates originating in other countries (Fig. 1). The high nucleotide sequence identity (> 99.8%) among the strains isolated in 1998 indicates that they were from a single origin and further spread to different townships, such as Pingtung (ID#98TW358). The only 1998 imported DENV-3 isolated from a traveler who had recently visited Indonesia was more closely associated with the genotype II isolates from Myanmar and older isolates from Thailand. This virus differed from the virus isolated during the 1998 Tainan outbreak, which might suggest that multiple genotypes of DENV-3 circulated in Indonesia. This observation is consistent with a previous study indicating that at least two subtypes of DENV-3 were present in Indonesia [18]. The phylogenetic analysis also suggested that a single 1999 isolate (ID#99TW628) from the same location as the 1998 epidemic was grouped together with the genotype III Sri Lanka isolates. Additional DENV-3 isolates from the first DENV-3-caused DHF outbreak in Taiwan (1994–1995) were grouped into genotype I. All these results implicated that repeated introductions of different genotypes of DENV-3 into Taiwan since 1994 were important causes of dengue epidemics, and that DENV-3 was not endemic in Taiwan. This situation may be similar in the subtropical region of China. Our country initiated airport fever screening during the severe acute respiratory syndrome (SARS) outbreak in 2003–04, and it successfully identified 40 confirmed, imported dengue cases [22]. Airport fever screening can thus quickly identify imported dengue cases, and may prevent a significant number of dengue outbreaks that would have been initiated by imported index cases. However, its cost-effectiveness in preventing any dengue epidemics in Taiwan will need to be evaluated in the future.

With different DENV-3 genotypes imported into Taiwan from Southeast Asia and other parts of the world, this virus collection provides an excellent opportunity to examine the sequence diversity of different genes of the full-length DENV-3 viral RNA genome for genotypes other than genotype IV. The highest p-distance of nucleotide diversity of the full-length genomes occurred for the NS2A gene (5.84% ± 0.54%), followed by the E gene (5.04% ± 0.32%). In contrast, the highest p-distance of amino acid diversity of the full-length genomes occurred for the capsid gene (3.13% ± 0.96%), followed by the NS2A gene (2.57% ± 0.62%). This observation is consistent with the previous analysis in DENV-1, DENV-3 and DENV-4 [16,34], although the precise cause of the increased rate of amino acid change in the NS2A gene is unknown. A similar observation could also be made while analyzing the full genomic sequences of West Nile virus (WNV) isolated from different animal species [35]. The flavivirus NS2A, a protein important for viral replication and particle formation [12], is cleaved by viral serine protease. A mutation at the basic P1 cleavage site residue in NS2A blocks this processing event and is lethal for virus production while still allowing RNA replication [36,37]. Furthermore, this basic residue in NS2A and an acidic residue in NS3 are important determinants for virus assembly and/or release [38]. Although the relative high sequence diversity of the NS2A gene of DENV-3 may be due to the lesser structural constraint required for NS2A, it is possible that positive selection pressures may be exerted on this gene. Especially in light of recent studies, NS2A together with NS4B and NS4A were identified as dengue virus-encoded proteins that could antagonize the interferon (IFN) response during viral infection [39,40]. Our analysis didn't detect any selection pressure exerted on the NS2A gene probably due to the small sample size; future studies will be needed to focus the selection pressure analysis on non-structural proteins and DENV evolution.

Several evolutionally conserved amino acid changes are preserved, which are unique in different DENV-3 genotypes (Table 3). These substitutions resulted in changes of its polarity, hydrophobicity or charge. Especially notable was the change from L to S at position 178 of the NS1 region, which is an amino acid substitution unique to genotype II. This might be the result of positive selection within the lineage of genotype II but not other genotypes. All DENV-3 isolates from Thailand belong to genotype II, and interestingly, based on a previous publication [16], strains of DENV-3 isolated prior to 1992 in Thailand may have been replaced by two new locally evolving strains. This could be a sign of a new genotype evolving in Thailand; however, most of the mutations or substitutions occurring were deleterious and a purifying selection of DENV-3 was suggested [16]. It is very likely that the previous analysis focused on only the E protein gene. Determining the possibility of a positive natural selection site in the non-structural genes of the new Thailand lineage will require further study. A number of T- and B-cell epitopes are present on the non-structural proteins, especially the NS1 gene [41-43]. Even though the biological significance of the L to S change at position 178 of the NS1 region is unclear, growing evidence supported by *in vitro *and *in vivo *studies suggest that there are certain evolutionary forces acting on the NS1 gene shaping the gene flow of the dengue viral population, which might differ during viral replication in mammalian and mosquito cells [44,45]. This is the first time that a positive selection pressure site was detected in a non-structural protein in DENV-3 and its importance together with its functional relevance to epidemic severity will need to be examined with a larger sample size.

The global distribution of different genotypes of DENV-3 indicates that they originated in Southeast Asia; these genotypes demonstrated higher epidemic potential with regards to severe DHF epidemics in Sri Lanka, Central and South America [46,47]. Genotype III, once its transmission cycle was established locally, soon resulted in DHF epidemics regardless of an increase in virus transmission or a change in circulating serotypes [7,14], supporting the hypothesis that virus strain is an important risk factor for DHF [48,49]. Two sub-lineages (isolated before and after 1989) existed within the DENV-3 genotype III strains from Sri Lanka, and the viruses isolated after 1989 were associated with the DHF epidemic [7]. We found that the strain isolated in 1999 from a indigenous dengue patient (99TW628) that did not lead to a large-scale epidemic of DF or DHF was more closely related to the lineage of the DENV-3 genotype III Sri Lankan strain isolated before 1989. Similarly, in Indonesia two sub-lineages of DENV-3 were present (isolated before and after 1998), and a greater DHF epidemic, especially in adult cases, was caused by the DENV-3 strains isolated after 1998 [50]. The DENV-3 strain isolated in Taiwan during the DHF outbreak in 1994 was actually more closely related to the old Indonesian strain of genotype I from 1976–78. While it is currently unknown how the different sub-lineages within each genotype are associated with different DHF epidemic potential, a recent publication suggested that changing serotype prevalence could lead to differential susceptibility to cross-reactive immune responses [16]. Furthermore, Wearing et al suggested that both vector and short-termed host cross-immunity are two factors responsible for dengue epidemics [51]. It would be necessary to strengthen comprehensive dengue virological surveillance, especially in those endemic and hyper-endemic areas/countries, to monitor the emergence of DENV strains with epidemic potential for better epidemic prevention and vaccine development.

## Competing interests

The authors declare that they have no competing interests.

## Authors' contributions

DYC and CCK designed and performed all the experiments and drafted this manuscript together. DYC participated in the sequence alignment and statistical analysis. JHH and YCW helped with collecting field human isolates and LJC helped with sequencing experiments, together. THL helped for the field mosquito collection and GJC formulated the idea for this study and also provided critical comments regarding this manuscript. All authors read and approved the final manuscript.
